# Effect of nutrient deficiencies on *in vitro *T_h_1 and T_h_2 cytokine response of peripheral blood mononuclear cells to *Plasmodium falciparum *infection

**DOI:** 10.1186/1475-2875-9-162

**Published:** 2010-06-14

**Authors:** Erasto V Mbugi, Marjolein Meijerink, Jacobien Veenemans, Prescilla V Jeurink, Matthew McCall, Raimos M Olomi, John F Shao, Jaffu O Chilongola, Hans Verhoef, Huub FJ Savelkoul

**Affiliations:** 1 Cell Biology and Immunology Group, Wageningen University, The Netherlands; 2 Host-Microbe Interactomics, Wageningen University, The Netherlands; 3 Danone Research, Wageningen, The Netherlands; 4 Department of Medical Microbiology, Radboud University, Nijmegen, The Netherlands; 5 Kilimanjaro Christian Medical Centre (KCMC), Moshi, Tanzania; 6 London School of Hygiene and Tropical Medicine, Nutrition and Public Health Intervention Research Unit, London, UK; 7 Muhimbili University of Health and Allied Sciences, Biochemistry Department, School of Medicine, Dar es Salaam, Tanzania

## Abstract

**Background:**

An appropriate balance between pro-inflammatory and anti-inflammatory cytokines that mediate innate and adaptive immune responses is required for effective protection against human malaria and to avoid immunopathology. In malaria endemic countries, this immunological balance may be influenced by micronutrient deficiencies.

**Methods:**

Peripheral blood mononuclear cells from Tanzanian preschool children were stimulated *in vitro *with *Plasmodium falciparum*-parasitized red blood cells to determine T-cell responses to malaria under different conditions of nutrient deficiencies and malaria status.

**Results:**

The data obtained indicate that zinc deficiency is associated with an increase in TNF response by 37%; 95% CI: 14% to 118% and IFN-γ response by 74%; 95% CI: 24% to 297%. Magnesium deficiency, on the other hand, was associated with an increase in production of IL-13 by 80%; 95% CI: 31% to 371% and a reduction in IFN-γ production. These results reflect a shift in cytokine profile to a more type I cytokine profile and cell-cell mediated responses in zinc deficiency and a type II response in magnesium deficiency. The data also reveal a non-specific decrease in cytokine production in children due to iron deficiency anaemia that is largely associated with malaria infection status.

**Conclusions:**

The pathological sequels of malaria potentially depend more on the balance between type I and type II cytokine responses than on absolute suppression of these cytokines and this balance may be influenced by a combination of micronutrient deficiencies and malaria status.

## Background

Frequent or chronic exposure to *Plasmodium falciparum *infection is thought to be a key element to immune protection against malaria in endemic areas [1]. Although the human immune system can kill parasites, it can also contribute to severe disease if not regulated and controlled to optimal levels [2,3]. In African countries, micronutrient deficiencies are common and may modulate immunity and predispose to infections. This is particularly relevant for young children who are most at risk of both malaria and micronutrient deficiencies.

Deficiencies in mineral elements and vitamins can result in suppression of innate, T-cell mediated and humoral responses [4,5]. Coordinating these responses are the cytokines which are produced interactively by several types of immune cells [2,4]. The immune response to malaria is specific for individual developmental stages of the parasite, and the balance in production of pro-inflammatory and anti-inflammatory cytokines seems to be critical for prognosis [5,6]. Following presentation of malaria antigens by antigen-presenting cells including dendritic cells, macrophages and occasionally B cells, naïve T helper (Th) cells proliferate and differentiate into specific T_h_ cell subsets. The pattern of T_h_ cell types, and the associated cytokine profile, probably depends on the type of antigen-presenting cells and their cytokine milieu, and on regulatory T-cells that suppress the proliferation and activity of B cells and T_h_ cells by the production of IL-10 and transforming growth factor (TGF)-β. Imbalance in these responses can result in an inefficient adaptive immune response to clear infection, and may contribute to pathological consequences. Several reports [7-15] have indicated possible roles of micronutrients on immune responses but either they have focused on other infections than malaria, or their effects have been evaluated in individuals older than five years, the age with the highest vulnerability to malaria.

It is hypothesized that the adaptive cytokine response to *P. falciparum *is influenced by micronutrient deficiencies that result in an imbalance between T_h_1 cells, with interferon (IFN)-γ as a signature cytokine, and T_h_2 cells, characterized by the production of interleukin (IL)-4, IL-5 and to some extent IL-13. Peripheral blood mononuclear cells (PBMCs) were isolated from Tanzanian children aged 6-72 months, and assessed *in vitro *the cytokine responses of these PBMCs upon exposure to erythrocytes parasitized by *P. falciparum*. These responses differ between donors with and without micronutrient deficiencies and in addition, the magnitude of PBMCs cytokine responses depended on *P. falciparum *infection status of the child at the time of blood collection.

## Methods

### Study area and population

The field work for this study was conducted in a lowland area around Segera village (S 05° 19.447', E 38° 33.249'), Handeni District, north-eastern Tanzania, in May-July 2006. Malaria is highly endemic in this area, with virtually all infections being due to *P. falciparum*. The local population comprises mostly poor farmer families growing maize and cassava for subsistence use. The study was approved by both Ethics Review Committees in The Netherlands and Tanzania (for Tanzania ethics review bodies, the reference numbers for KCMC and National Ethics Review Committee were 094 and NIMR/HQ/R.8a/VolIX/540, respectively). Informed consent was obtained from community leaders and local government officials, and from parents or guardians.

### Sampling methods, eligibility criteria and preliminary laboratory analyses

The details of sampling method, field procedures, isolation of peripheral blood mononuclear cells (PBMCs) are provided elsewhere [16,17]. In brief, children aged 6-72 months were recruited in the study and were clinically examined before sample collection. Children were eligible to participate if they had no signs of severe febrile disease or severe malnutrition at the time of assessment. Dip stick test was used for diagnosis complimenting microscopy and providing a wide chance for detecting asymptomatic malaria infection [18,19]. Whole blood samples from the study children were collected after overnight fasting. PBMCs were isolated using Ficoll density gradient centrifugation. *P. falciparum*-parasitized and unparasitized erythrocytes were prepared as described elsewhere [20,21], and kept under frozen conditions until the stimulation experiments.

### Determination of plasma indicators of mineral element status

Plasma samples were diluted 20 times in milliQ [22], and concentrations of zinc, magnesium and copper were measured by inductively-coupled plasma atomic emission spectrometry (ICP-AES) (Vista Axial, Varian, Australia). To determine variability in outcomes, measurements were replicated five times: with mean values set at 100%, measurements varied between 97% to 102% for zinc, 99% to 102% for magnesium, and 97% and 102% for copper. Plasma concentrations of ferritin and C-reactive protein were measured as indicators of iron stores and inflammation, respectively by using a Behring nephelometer (BN ProSpec; Dade-Behring) in The Netherlands (Meander Medical Centre) and will be reported separately.

### PBMCs stimulation

PBMCs were cultured at 10^6 ^cells/well in sterile polystyrene 48-well plates with flat-bottom wells (Corning, Cat No. 3548, NY 1483, USA) in Yssel's culture medium [23], which is a modification of Iscove's modified Dulbecco's medium (IMDM),. The medium is recommended for the culture of cells growing in suspension, such as human T and B cell lines and is especially recommended for the generation and long-term culture of antigen-specific T cell and NK clones [24,25]. This culture thereby permits the outgrowth of all T-cell subsets induced by exposure to a complete malaria extract. Aliquots of *P. falciparum*-parasitized red blood cells (pRBC) were thawed, re-suspended in Yssel's^+ ^medium [17] with 2% human AB^+ ^serum plus 1% penicillin-streptomycin and 1% fungizone (Gibco-BRL, Invitrogen, Grand Island NY, USA), and added to PBMCs in a ratio of 2:1 (2 × 10^6 ^pRBC to 1 × 10^6 ^PBMC). PBMC were also cultured under similar conditions with unparasitized erythrocytes (uRBC)(2 × 10^6 ^cells/well) as a negative control, and with soluble antibodies to CD3 and soluble antibodies to CD28 (Cat. No.555336 and 555725, Becton-Dickinson Pharmigen, Alphen aan den Rijn, The Netherlands) as a positive control. Cell culture plates were incubated at 37°C in a humidified atmosphere containing 5% CO_2_. Based on previous studies [26-28] and our own preliminary experiments (Mbugi E, Meijerink M et al, unpublished data), seven days of continuous stimulation will be optimal for observing differences in PMBCs responses to exposure with non-parasitized and parasitized RBCs. Thus, after six days of culture, monensin was added to cells to allow for accumulation and subsequent staining of intracellular cytokines. At day 7 of culture, aliquots of supernatant were collected from parallel non-monensin treated cultures; concentrations of type I cytokines (IL-1β, IL-12p70, TNF, ILN-γ) and type II cytokines (IL-4, IL-5, IL-10, IL-13) in culture supernatants were measured using a Cytometric Bead Array System (FACSCanto, Becton-Dickinson).

### Proliferation and activity of leukocyte subsets

Proliferation assays were performed to determine the activity potential of cells and to determine whether selected individuals displayed intrinsic differences in their T-cell compartments. To distinguish PBMCs subsets, cultured cells (5 × 10^5^) were stained for 30 min, at 4°C in the dark with a combination of fluorophore-bound antibodies against CD4 (T helper cells), CD8 (cytotoxic T cells) and CD45 (all leucocytes) (Becton-Dickinson Pharmingen, Alphen aan den Rijn, The Netherlands). The cells were then centrifuged (500 × *g*, 5 min, 4°C), washed, re-suspended in PBS for subsequent staining for Ki-67 protein. This protein is present during all active phases of the cell cycle, but not in resting cells [29]. After CD marker staining, cells were fixed and permeabilised by incubation (15 min, 4°C, dark condition) with BD Cytofix/Cytoperm (catalogue no. 554722, Becton-Dickinson Pharmingen). The cells were subsequently washed twice with BD Perm/Wash buffer™ (catalogue no.554723, Becton-Dickinson Pharmingen), centrifuged (300 × *g*, 10 min, 4°C), re-suspended in BD Perm/Wash buffer and incubated with Ki-67 detection antibodies (catalogue no. 556026, Becton-Dickinson Pharmingen) (30 min, 4°C, dark conditions). Thereafter, the cells were washed twice with perm/wash buffer and suspended in PBS with counting beads for subsequent flow cytometry.

### Intracellular cytokine staining

Cultured cells (5 × 10^5^) were incubated (for 30 min, at 4°C in the dark) with 20% human AB serum in PBS to block Fc receptor binding and stained with antibodies against CD4 and CD25 to detect activated Th cells, centrifuged (500 × *g*, 5 min, 4°C), fixed, permeabilized and washed as described above, and re-suspended in PBS for subsequent intracellular staining for IL-10 and IL-4 using antibodies against these cytokines (BD Pharmigen, Alphen aan den Rijn, The Netherlands). After incubation with anti-IL-10 and anti-IL-4 detection antibodies, the cells were washed twice with perm/wash buffer and re-suspended in PBS for analysis by flow cytometry.

### Flow cytometry

Analyses were performed on a FACSCanto II flow cytometer and analysed with FACSDiva™ software (both Becton-Dickinson Biosciences).

### Statistical analysis

Data were entered and analysed using SPSS for Windows (version 15.0. SPSS Inc., Chicago, IL, USA). Zinc deficiency and low zinc status were defined as plasma zinc concentrations < 9.9 μmol/L and < 10.7 μmol/L, respectively; low magnesium status was defined by magnesium concentration < 750 μmol/L; iron deficiency anaemia was defined by coexisting iron deficiency (plasma ferritin concentration < 12 μg/L) and anaemia (haemoglobin concentration < 110 g/L). The association between inflammation (CRP levels) and sex, age, malaria status as well as nutritional status was determined by Fisher's Exact Test. Cytokine concentrations were log-transformed to obtain normally distributed values. Group differences were analysed assuming t-distributions, and associations between continuous variables were assessed using linear regression analysis. Effects of log-transformed data were expressed in their natural units by exponentiation, and reported as the percentage difference relative to the reference value. The analyses of the cytokine responses to pRBCs are reported. As expected, the average response to uRBCs (negative control) was less than to pRBCs, whereas the average response to CD3/CD28 (positive control) was higher. Correction for these responses does not change the estimates of the associations between nutrient status and cytokine responses, or between malarial infection status and cytokine responses.

## Results

### Study population and characteristics

The study population consisted of 304 children; 301 were within the eligible age range; for three children were found to be older but these were retained in the analysis. Characteristics of the study population and crude associations between malarial infection and nutrient markers are provided elsewhere [16]. In short, the following prevalence values were found: malarial antigen as assessed by dipstick test: 45.2%; low zinc status 63.1% (188); zinc deficiency: 48.3% (144); low magnesium status: 65.1% (194); iron deficiency anaemia: 9.4% (26); malaria: 46.1% (140). Malaria status at inclusion was found to associate with age and iron deficiency anemia, but not with zinc or magnesium deficiency [16]. There was no evidence that inflammation (determined by CRP levels) was associated with zinc deficiency, magnesium deficiency or iron deficiency anaemia although malaria and age seemed to be associated with inflammation (Table 1).

**Table 1:** Micronutrient status in relation to age, inflammation and *Plasmodium *infection

	Zinc deficient			Magnesium deficiency			Iron deficiency anaemia		
	
	Prevalence		p	Yes	No	p	Present	Absent*	p
**Age**			0.03			0.02			< 0.01
6-12 months	48%	[12/25]		60%	[15/25]		63%	[5/8]	
12-24 months	29%	[14/48]		48%	[23/48]		76%	[16/21]	
24-48 months	50%	[60/119]		66%	[78/119]		8%	[4/52]	
48-72 months	55%	[58/106]		74%	[78/106]		4%	[3/85]	
**Inflammation**			0.22			0.30			0.80
Present	43%	[43/100]		61%	[61/100]		14%	[5/35]	
Absent	51%	[101/197]		68%	[133/197]		18%	[23/130]	
***Plasmodium *infection**			0.49			0.63			< 0.001
Present	46%	[63/137]		66%	[107/161]		4%	[2/55]	
Absent	50%	[81/161]		64%	[87/137]		23%	[26/111]	

Values indicate prevalence [n/n]. P-values obtained by Chi-square test or Fischer's Exact test.

* Absence of iron deficiency defined as being non-anaemic and not being iron deficient. Total sample size was 304; summed numbers below this value is due to missing observations.

### Induction of cytokine production

Lower concentrations of cytokines were detected in uRBC than in pRBC indicating the differences in *in vitro *mitogenic activities on PBMC. Stimulated PBMC responded more strongly with IFN-γ production as compared to other cytokines. Production of IL-4 was lowest regardless of the micronutrient status. In general, the composition of cytokines in day 7 supernatants consisted of IFN-γ , TNF, IL-1β, IL-13, IL-10, IL-12p70, IL-5 and IL-4, in declining order of concentration.

### Proliferation and intracellular cytokine staining

**Figure 1:**
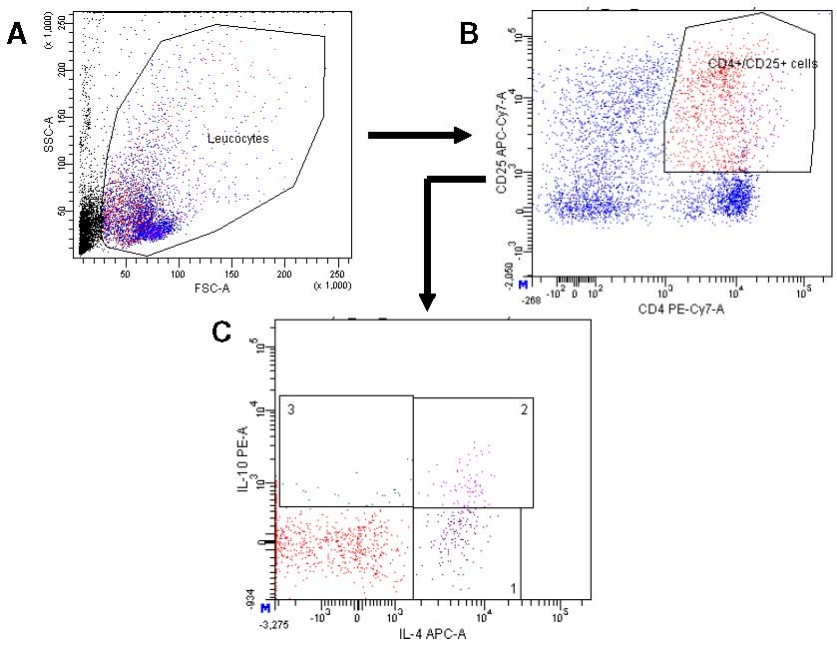
**Representative example of the flowcytometric analysis of the IL-10 secreting CD4^+^CD25^+ ^population of PBMCs stimulated with malaria extract for 7 days**. A. Leukocytes were identified by forward (FSC) and sideward (SSC) scatter. B. Of these leukocytes staining was performed with CD4-PE-Cy7-A and CD25-APC-Cy7-A labeled monoclonal antibodies from BD Pharmingen. C. The CD4^+^CD25^+ ^double positive cells were stained intracellularly with IL-4 APC-A and IL-10 PE-A labeled monoclonal antibodies from BD Pharmingen. Panel 1 contains 14% IL-4 single positive CD4^+^CD25^+ ^cells, panel 3 contains 2.5% IL-10 single positive CD4^+^CD25^+ ^cells, and panel 2 contains 7.2% IL-4^+^IL-10^+ ^double positive CD4^+^CD25^+ ^cells.

The average proportion of malaria extract-specific proliferating cytotoxic T-cells (CD8^+ ^Ki67^+^) and proliferating T_h _cells (CD4^+ ^Ki67^+^) relative to the general proliferating leucocytes were 4% and 21%, respectively. In Figure 1, a representative example of a flow cytometric analysis of a malaria-specific CD4^+ ^T-cell response in the PBMCs of a malaria-infected child is shown. Of the leukocytes responding to the malaria extract after seven days of culture, on average 20% of the leukocytes were activated Th cells (CD4^+ ^CD25^+^; 23% in Figure 1), part of them may be naturally occurring or probably inducible regulatory T-cells (Tr). Intracellular cytokine staining revealed that the proportion of CD4^+^/CD25^+ ^cells producing both IL-4 and IL-10 (average 19%; 7.2% in Figure 1) was higher than cells producing only IL-10 (average 4%; 2.5% in Figure 1) or only IL-4 (14% in Figure 1). This indicated that most anti-inflammatory cytokine response came from IL-4^+^IL-10^+ ^double producing cells, rather than from single IL10^+ ^cells.

### Influence of nutrient deficiencies on cytokine responses to stimulation with malaria parasites

The association between nutrient deficiencies and cytokine responses was determined assuming no interaction with malarial infection. The effect change in cytokine concentration under different conditions of micronutrients status is shown in Tables 2 and 3, respectively. For this analysis only the data of individuals of which the PBMCs cultures stimulated by malaria extract yielded detectable cytokine levels that are indicative of malaria-specific responding T-cells by induced proliferation and cytokine synthesis were used. Thus the number of individuals differs for every individual cytokine analyzed. Overall, zinc deficiency was associated with increased supernatant concentrations of TNF and IFN-γ (by 37% and 74%, respectively), and seemed associated with increased concentrations of IL-5 and IL-13 (Tables 2 and 3). Magnesium deficiency was associated with an 80% increase in IL-13 concentrations, and seemed associated with a 49% increase in IL-5 concentrations. Iron deficiency anaemia was associated with increased concentrations of IL-12 by 37%, and seemed associated with a 34% decrease in IL-5 concentrations.

**Table 2:** Associations between nutrient deficiencies and type I cytokine responses to *in vitro *stimulation of PBMCs with malaria-parasitized red blood cells

	Supernatant concentration (ng/L) after 7 days of stnimlation			
	
Nutrient status	IL-12	TNF-α	IFN-γ	IL-1b
Zinc				
Deficient	2.4 (19)	4.3 (35)	46.6 (42)	4.5 (33)
Replete	2.4 (13)	3.2 (28)	26.8 (37)	5.5 (27)
Effect	-1% [-29% to 38%]	37%[14% to ll8%]	74% [24% to 297%]	-19% [-47% to 26%]

Magnesium				
Deficient	2.4 (23)	3.7 (43)	34.1 (56)	4.9 (41)
Replete	2.3 (9)	3.9 (20)	41.1 (23)	4.8 (19)
Effect	4% [-28% to 51%]	-6% [-43% to 57%]	-17% [-66% to 108%]	3% [-36% to 64%]

Iron deficiency anaemia

**Table 3:** Associations between nutrient deficiencies and type II cytokine responses to *in vitro *stimulation of PBMCs with malaria-parasitized red blood cells

	Supernatant concentration (ng/L) after 7 day of stimulation		
	
Nutrient status	IL-5	IL-10	IL-13
Zinc			
Deficient	6,6(15)	3.2 (29)	10.0(31)
Replete	5.2(14)	3.4 (26)	7.3 (28)
Effect	26% [-54% to 146%]	-6% [-3 5% to 35%]	3 7% [-41% to 2 19%]

Magnesium			
Deficient	6,5 (22)	3.4 (40)	10.0 (44)
Replete	4.3 (7)	3.2(15)	5.6(15)
Effect	49% [-54% to 382%]	5% [-30% to 58%]	80% [31% to 371%]

Iron deficiency anaemia			
Yes	4.63 (15)	3.51 (26)	8.30 (20)
No	7.01 (15)	3.35 (30)	9.32(41)
Effect	-34% [-75% to 74%]	5% [-27% to 50%]	-11% [-60% to 100%]

Values indicate geometric means (n) or effect [95% CI]

### Interaction between nutrient deficiencies and malarial infection on cytokine response to stimulation

**Figure 2:**
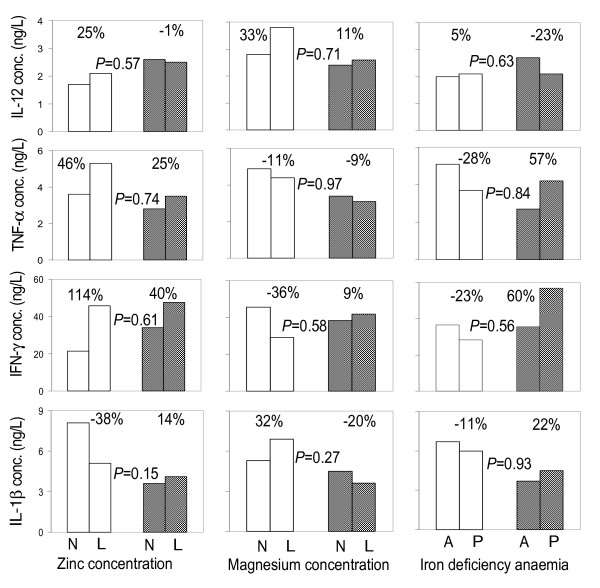
**Associations between micronutrient status and supernatant type I cytokine concentrations following 7 days of PBMCs stimulation with *Plasmodium falciparum*-infected erythrocytes, by malaria infection status of the child at the time of blood collection**. N: Normal concentrations; L: low concentrations; A: absent; P: present. Percentages indicate paired group differences in cytokine concentrations. Data from children without and with malaria infection at the time of blood collection are indicated with open and shaded bars, respectively. *P*-values indicates the interaction between nutrition and malaria infection status.

**Figure 3:**
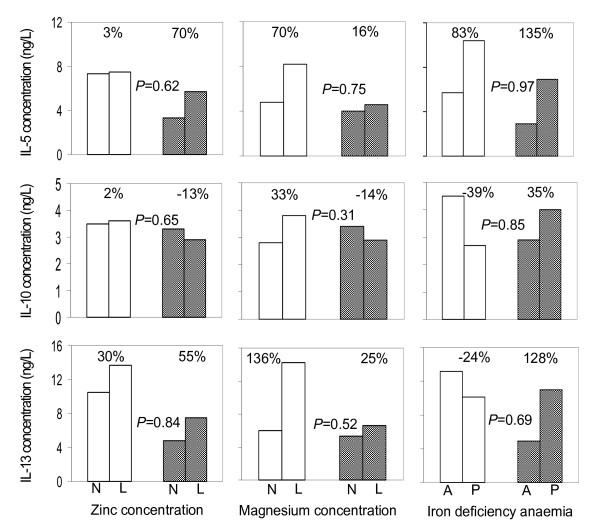
**Associations between micronutrient status and supernatant type II cytokine concentrations following 7 days of PBMCs stimulation with *Plasmodium falciparum*-infected erythrocytes, by malaria infection status of the child at the time of blood collection**. N: Normal concentrations; L: low concentrations; A: absent; P: present. Percentages indicate paired group differences in cytokine concentrations. Data from children without and with malaria infection at the time of blood collection are indicated with open and shaded bars, respectively. *P*-values indicate the interaction between nutrition status and malaria infection status (nutrient vs. malaria).

In some cases, malarial infection at the time of blood collection seemed to influence the associations between nutrient deficiencies and cytokine responses to stimulation. For example, in children without malaria, zinc deficiency was associated with an increase in supernatant concentration of IFN-γ by 114%; 95% CI: 41% to 677% as compared to an increase of 40%; 95% CI: -53% to 314% in their peers with malaria infection. Similarly, in children without malarial infection, iron deficiency anaemia was associated with a decrease in IFN-γ concentration by 23% (95% CI: -78% to 177%) as compared to a 60% increase (95% CI: 45% to 368%) in their peers with malaria infection. In none of these cases, however, was such interaction supported by statistical evidence, as indicated by the high *P*-values in Figures 2 and 3. In other words, there is no evidence that the relationships between nutrient markers and cytokines depend on malarial infection. This reflects that nutritional deficiencies association with *in vitro *cytokine responses is independent of malaria status at time of blood collection.

### Relationship between IFN-γ and type II cytokines

**Figure 4:**
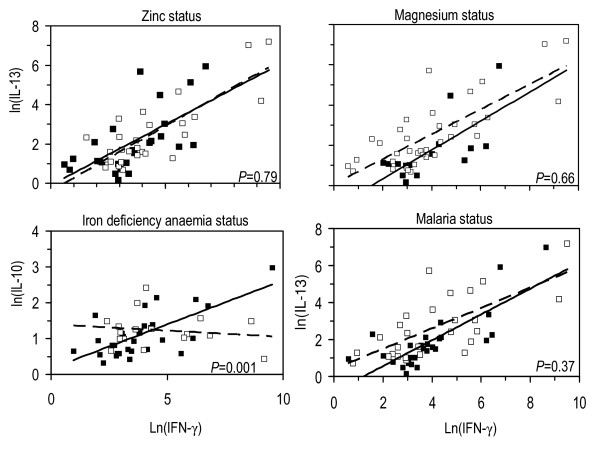
**Relationships between supernatant concentrations of IFN-γ, IL-10 and IL-13 under different conditions of micronutrient and malaria status at the time of blood collection, following 7 days stimulation of PBMCs with *Plasmodium falciparum*-infected erythrocytes**. Regression lines and dots in respective panels: closed dots and solid lines = zinc replete, magnesium replete, no iron deficiency anaemia and no malaria; open dots and dashed lines = zinc deficiency, magnesium deficiency, iron deficiency anaemia and positive malaria tests at time when blood was collected. The differences in slopes for other relationships are shown in Table 3. *P*-values have been calculated to indicates whether the interaction between nutrition status and malaria infection status have impact on the association between IFN-γ and IL-10.

The influence of nutrient deficiencies on the relationships between IFN-γ and some type II cytokines was assessed (Table 4, Figure 4). Overall, there was a clearly detectable positive linear relationship between IFN-γ and IL-5, IL-10 and IL-13. There was weak evidence however, that the slopes of regression lines differ with zinc, magnesium or malaria infection status. On the other hand, there was strong evidence that the slopes of regression lines for the association between IFN-γ and IL-10 differed with iron deficiency anaemia status (*P *= 0.001). The change in slopes in the latter relationship was such that in iron deficiency anaemia there existed a negative linear relationship signifying that an increase in IFN-γ lead to a decrease in IL-10. There was no evidence that the slopes of regression lines for the association between IFN-γ and IL-10 differed (Figure 4) in zinc deficiency, magnesium deficiency and malaria infection. However, the results indicate that a smaller increase in concentration of IL-10 was associated with a relatively larger increase on IFN-γ (slopes). Subsequent analysis on whether deficiencies and malaria infection status influence the association among T_h_1 and T_h_2 cytokines, apart from IFN-γ (data not shown) revealed an overlap in slopes of linear associations. There was weak evidence that magnesium deficiency, zinc deficiency and malaria infection at time of blood collection influenced these associations.

**Table 4:** Effect change in linear relationships and differences in slopes of supernatant concentrations of cytokines measured after 7 days of stimulation with malaria antigens in children with different nutritional and malaria status at time when blood was collected.

Predictors	Cytokine pairs	Change in slope	95% CI
Zinc	IFN-γ vb IL-5	32%	-62% to 357%
Zinc	IFN-γ vsIL-10	-12%	-43% to 35%
Zinc	IFN-γ vsIL- 13	12%	-52% to 157%
Magnesium	IFN-γ vs IL-5	-38%	-89% to 256%
Magnesium	IFN-γ vsIL- 10	-36%	-61% to 5%
Magnesium	IFN-γ vsIL- 13	-20%	-71% to 118%
IDA	IFN-γ vs IL-5	-45%	-81% to 58%
IDA	IFN-γ vsIL- 10	-48%	-63% to -36%
IDA	IFN-γ vsIL- 13	-26%	-66% to 58%
Malaria	IFN-γ vs IL-5	174%	-5% to 689%
Malaria	IFN-γ vsIL- 10	-23%	-17% to 83%
Malaria	IFN-γ vsIL- 13	40%	-34% to 197%

IDA: Iron deficiency anaemia

## Discussion

### Cytokine production

We have revealed a cytokine concentration of IFN-γ, TNF, IL-1β, IL-13, IL-10, IL-12, IL-5 and IL-4 in declining order following a 7 days *in vitro *stimulation of PBMCs using pRBCs (Tables 1 and 2). The cytokine concentration in the supernatant following *in vitro *stimulation cannot be extrapolated to number of responding cells but at least hints on what could happen *in vivo *amid natural infection. The relatively low concentrations of IL-12, IL-5 and IL-4 may reflect that probably these cytokines are needed in very minute amounts, present only very temporarily or are gradually degraded or consumed by cells earlier after response to infection. It was anticipated that these cytokines are more active earlier than seven days. Comparably, in both zinc replete and zinc deficient group cells seemed to respond better towards production of IFN-γ, TNF, IL-1β, IL-13 and IL-10 than other cytokines *in vitro*. This might mean that these cytokines are crucial for continued elimination of the parasite at different stages of infection (pre-erythrocytic and erythrocytic stages) *in vivo *although this hypothesis may be unjustifiable based only on *in vitro *data.

### Effects of zinc and other micronutrients on cytokine production

In malaria endemic areas, repeated exposure to infection by *P. falciparum *results into naturally acquired immunity that fails to develop in areas where malaria is hypoendemic, epidemic or mesoendemic. This means that the potential mechanisms of protection and immunological memory depend among other factors, on the degree of exposure and pattern of malaria transmission [30]. Regardless of age, immunity to malaria is generally low in populations living in areas with low or unstable transmission. In such a situation, clinical malaria and possibly severe complications can occur in both children and adults [31]. Although it appears not to be sterile, immunity to malaria is protective provided there is a constant exposure to infection and may be strengthened by good nutrition. This study provides *in vitro *results on the effect of some nutrients on the mediators of immune response to malaria in Tanzanian children by using intact *P. falciparum *parasitized erythrocytes (pRBC) to induce immuno-regulatory cytokines [26,32] reflecting the real *in vivo *situation. Among nutrients explored in this study, zinc, magnesium and iron deficiency anaemia was associated with variable concentrations of one or more cytokines from both T_h_1 and T_h_2 groups, which mediate the immune response to malaria. Prasad [33] reported zinc deficiency to cause an imbalance between T_h_1 and T_h_2 functions in an experimental human model in which production of IFN-γ (product of T_h_1) was decreased and that no effects were predictable in production of IL-4 (and IL-10) (products of T_h_2). These findings contrast with previous findings by Prasad in the sense that zinc deficiency was associated with higher levels of IFN-γ, TNF and IL-12 (Figure 2), but concur with the findings on IL-10 (Figure 3). This is especially intriguing as it may imply that in zinc deficiency, the immune response to malaria shifts to more cellular-mediated immune response before tailing off. Previous results [13,34,35] have indicated that zinc deficiency is associated with a decreased ratio of CD4^+ ^to CD8^+ ^cells and is indicative of cytotoxic immune response. It could be that, in this study, of the activated CD4+ T-helper, T_h_1 cells were dominant in producing type I cytokines in cells from zinc deficient children. Our study partly agrees with available reports that zinc deficiency affects both cell-mediated immune responses and humoral responses [14] and that B cell proliferation is less dependent on zinc, albeit zinc deficiency may result in fewer naïve B cells for production of antibodies to new antigens [36].

Magnesium deficiency was associated with an increase in the concentration of IL-13 among type II cytokines. Little work has been done on the role of magnesium in immune response to malaria and our results draw attention to the role of magnesium in cytokine production in reaction to malaria infection. Report [37] indicates that IL-13 and IL-4 are major cytokines driving the polarization of the immune response towards T_h_2. IL-13 is also believed to regulate immunoglobulin switching from IgG isotype to IgE, this is particularly important because it signifies that prolonged magnesium deficiency may predispose individuals to hypersensitivity reactions. Type I cytokines dominate in cellular immune responses while type II cytokine dominance implies humoral immune response [38]. The findings may reflect that magnesium deficiency is associated with an increase in IL-12 and IL-1β but these responses become weakened in malaria infection. On the other hand, the increase in IL-12 and IL-1β concentrations in zinc deficiency further go up in malaria infection (Figure 2) which may imply that in zinc deficiency the potential for production of pro-inflammatory cytokines following malaria infection is high, rising the risk for development to pathology. To the contrary, in malaria-infected, magnesium deficient children, the concentrations of IL-12, IL-1β (type I) and IL-5, IL-10 and IL-13 (type II cytokines) do not increase to levels attained in uninfected peers (Figures 2 and 3). In other words, the increase in cytokine concentrations due to magnesium deficiency in malaria infection does not compensate for that observed in uninfected. Thus, although in zinc deficiency, the immune system is more likely to use cellular responses as a weapon to fight against malaria opting to antibody responses in case of magnesium deficiency, the responses in magnesium deficiency might be weaker than in zinc deficiency.

The role of iron in the induction of a protective immune response is still debatable. The findings reported here indicate variable effects of iron deficiency anaemia on cytokine concentration. While levels of TNF, IFN-γ and IL-1β (type I) and IL-10 and IL-13 (type II) seemed decreased in iron deficiency anaemia, the levels of IL-12 and IL-5 appeared increased (Figure 3). These variable effects of iron deficiency on a range of both type I and type II cytokines are critical as these may lead to unstable cytokine response failing to inhibit the parasite. Reports on iron nutrition in children living in malaria endemic areas have indicated some association between IL-4 with all biochemical indices of iron [39]. In this study that was carried on the coast of Kenya, authors also report an increase in IL-10 serum mRNA expressions in malaria blood-smear positive children, results which are concordant with our *in vitro *results on iron deficiency anaemia despite the weak evidence.

### Malaria infection status and the profile of cytokine production under conditions of nutrient deficiencies

Comparing within groups our findings show an increase in type I cytokines (TNF, IFN-γ and IL-1β) in association with malaria infection in zinc deficient children as compared with zinc sufficient individuals, although this increase is less than amongst uninfected donors. However, an increase in IL-12 concentration seems to be independent of zinc deficiency (Figure 2) signifying that malaria infection is associated with induction of increased IL-12 production independent of zinc status. The levels of IL-12 at day 7 of stimulation support our previous results (Mbugi et al, submitted) that in malaria infection, IL-12 is produced later than 24 hrs of stimulation. With slightly decreased levels of IL-10 in association with malaria infection in zinc deficient children, the findings possibly reflect that zinc deficiency primarily results in pathological consequences of type I cytokines due the reduced regulatory role of the cytokine IL-10. In magnesium deficiency, malaria infection were associated with both type I and type II responses but the increase is not sufficient to compensate for the levels attained in malaria negative individuals regardless of magnesium status (Figures 3 and 4). These results may reveal that magnesium deficiency can lead to immune incompetence in response to malaria infection.

Iron deficiency anaemia appeared to induce a similar increased trend in both types of cytokine amongst malaria infected donors. The only exception was IL-12, which was reduced in association with iron deficiency anaemia although the levels were higher than those attained in children without malaria infection. The high levels of IFN-γ in iron deficiency anaemia may be an indication that protection from clinical malaria reported in iron deficiency [39,40] is probably through cell mediated immune responses. Interestingly, with the exception of IL-12, this study found an increase in both type I and type II cytokines in association with iron deficiency anaemia in children with malaria infection (Figures 2 and 3). Available report [41] have speculated about the role of iron deficiency in limiting the severity of the inflammatory response. The argument corresponds with our findings and it could be a result of increased secretion of anti-inflammatory cytokine in response to increase in levels of pro-inflammatory cytokines in malaria infection. However, the observation that the increase in cytokine production could not reach the levels in children without malaria may be due to a combined effect of nutrients deficiencies other than iron in co-existence. It is possible that in addition to the depletion of iron to the parasite that may occur in iron deficiency anaemia the host cells, including immune cells, are also depleted of iron [42] thus reducing the capacity for sufficient cytokine production.

The modulation of immune response by iron rests on its effects on the function of T_h_1 mediated response and supply of this nutrient to the parasite [43], in particular, withdrawal of iron is said to increase T_h_1 mediated immune function *in vivo *[42]. This study found an association of iron deficiency anaemia with slightly reduced concentrations of IL-1β, IFN-γ and TNF in children without malaria but an increment in children with malaria infection. This reflects that iron deficiency anaemia may be associated with increase in concentration of Type I cytokines in malaria infection. Iron deficiency anaemia is thus most likely associated with variable effects of both type I and type II cytokine responses (Figures 2 and 3) rather than the reported discriminate effects between the two arms [44].

### Linear association between type I and type II cytokines

Associations between cytokine production under different conditions of nutrients and malaria status may be predictive for disease outcome. We found relationships between type I and type II cytokines in micronutrient deficient and replete groups and they were variably influenced by the malaria status (Table 3). However, the significant difference in slopes in the association between IFN-γ and IL-10 with respect to iron deficiency anaemia status, in particular the negative association seen in iron deficiency anaemia, emphasizes that probably the response shifts in deficiency situations from one type of cytokine response to the other; particularly the balance between IFN-γ and IL-10 which is said to be critical in controlling malaria infection. This underscores the notion that micronutrients may have no grossly visible effects under normal situations but they do when the body is destabilized in terms of immune protection during infections. To emphasize, there is strong evidence that the association between IFN-γ and IL-10 is influenced by iron deficiency anaemia: in children without iron deficiency anaemia, IFN-γ responses are positively associated with IL-10 responses, whereas this association seems absent or weakly negative in children with iron deficiency anaemia (lower-left panel of Figure 3). This seems supported by weak evidence of similar interaction in the same direction when examining the influence of iron deficiency anaemia on the association between IFN-γ and IL-5, and between IFN-γ and IL-13 (Table 3). These associations may reveal that the regulatory T-cell responses (and possibly the T_h_2-responses) in malaria are suppressed in iron deficiency anaemia. In addition, there is substantial evidence that the relationship between IFN-γ and IL-5 is influenced by the presence of malarial infection at the time of blood collection: the association between the responses in IFN-γ and IL-5 is steeper in children with malarial infection than in their peers without infection (Table 3); this seems supported by weak evidence of similar interaction in the same direction between IFN-γ and IL-13 (Table 3). These data provide evidence that in malaria, previous malarial infection suppresses the T_h_2 (regulatory) responses to the disease. As regards to zinc and magnesium deficiencies, there is no evidence that the respective deficiencies influence the associations between IFN-γ and T_h_2 cytokines (Table 3). However, the findings that nutritional deficiencies and malaria status at time of blood collection are variably associated with T_h_2 responses independent of IFN-γ alerts to the importance of nutritional component in boosting immune response to malaria.

Not only zinc deficiency results into significant impact on cytokine responses to infections [7,9,14,45-48] but also other nutrients, like magnesium and iron. The results show that zinc deficiency may have more impact on type I cytokine responses while magnesium has selective effects on type II responses. In addition, the results also seem to indicate that in iron deficiency anaemia, the prevalent cytokine response is more of type I than type II responses. A recent randomized controlled trial conducted in Burkina Faso has suggested that a combined vitamin A plus zinc supplementation reduced the risk of fever and clinical malaria episodes among children aged 6 to 72 months [49], and this combination may be included in control strategies to fight against malaria in African children. However, it does not exclude the contribution of other micronutrients that have not been reported in this paper which need to be further explored.

This study has shown weak evidence [50] of effect of nutrients deficiencies on association between cytokine concentration and malaria status at time of blood collection. This could be due to small sample size to detect differences; the use of confidence intervals in our analysis however, gives a strong reflection of what could be happening as it shows a range within which the true effect is likely to lie. A larger sample size could allow detection of even minor differences leading to a proposal to a larger study particularly in the intervention study. The protective immune response to malaria is said to target a broad antigenic repertoire that go beyond parasitic developmental stages [51]. Here, parasitized erythrocytes were used to induce cytokine response in PBMCs providing intimation that sterile immune protection focusing on whole parasite vaccines could be rewarding [52].

## Conclusions

In conclusion, micronutrient deficiencies may be variably associated with impaired cytokine production. Zinc deficiency and iron deficiency anaemia have shown to be associated with remarkable increases in type I cytokine production, implying a shift in the balance of the immune towards pro-inflammatory and cellular type in these conditions. It may mean that zinc deficiency and iron deficiency anaemia directly induce increased production of pro-inflammatory cytokines or causes an imbalance in regulatory anti-inflammatory cytokines as reflected by increased pro-inflammatory cytokines. Since these pro-inflammatory cytokines have been associated with pathological consequences like cerebral malaria, it should be further assessed to what extent supplementation with zinc and iron is beneficial in children with deficiencies for these nutrients. Consideration of micronutrient supplementation may also be of value if incorporated in vaccine programs in endemic areas to boost immune responses to malaria.

## Conflict of interest

The authors declare that they have no competing interests.

## Authors' contributions

EVM: participated in protocol development, laboratory analysis, drafting and writing the manuscript; MM: participated in protocol development, designing and conducting laboratory analysis; JV: conducted the field work, assisted in protocol development, laboratory analysis and manuscript preparation; MMcC and JOC: assisted in protocol development, laboratory work and interpretation of data; JFS and RMO: co-directed the field work; HV: conceived the study, acquired funds, directed the field work, co-supervised data analysis and manuscript preparation; HFJS: assisted in study design, acquired funds, directed the study, supervised protocol development and data analysis. All authors have read and approved the final manuscript.

## References

[B1] Aultman KS Gottlieb M Giovanni MY Fauci AS Editorial: *Anopheles gambiae *genome: completing the malaria triad Science 2002 298 13 10.1126/science.298.5591.13 12364752

[B2] Riley EM Wahl S Perkins DJ Schofield L Regulating immunity to malaria Parasite Immunol 2006 28 35 49 10.1111/j.1365-3024.2006.00775.x 16438675

[B3] Perlmann P Troye-Blomberg M Malaria and the immune system in humans Chem Immunol 2002 80 229 242 full_text 12058641 10.1159/000058846

[B4] Riley EM Is T-cell priming required for initiation of pathology in malaria infections? Immunol Today 1999 20 228 233 10.1016/S0167-5699(99)01456-5 10322302

[B5] Stevenson MM Riley EM Innate immunity to malaria Nat Rev Immunol 2004 4 169 180 10.1038/nri1311 15039754

[B6] Torre D Speranza F Martegani R Role of proinflammatory and anti-inflammatory cytokines in the immune response to *Plasmodium falciparum *malaria Lancet Infect Dis 2002 2 719 720 10.1016/S1473-3099(02)00449-8 12467687

[B7] Fraker PJ King LE Reprogramming of the immune system during zinc deficiency Annu Rev Nutr 2004 24 277 298 10.1146/annurev.nutr.24.012003.132454 15189122

[B8] Fraker PJ Roles for cell death in zinc deficiency J Nutr 2005 135 359 362 15735063 10.1093/jn/135.3.359

[B9] Fraker PJ King LE Laakko T Vollmer TL The dynamic link between the integrity of the immune system and zinc status J Nutr 2000 130 1399 1406 10.1093/jn/130.5.1399S 10801951

[B10] Shah D Sachdev HP Effect of gestational zinc deficiency on pregnancy outcomes: summary of observation studies and zinc supplementation trials Br J Nutr 2001 85 Suppl 2 101S 108S 10.1079/BJN2000301 11509097

[B11] Shankar AH Genton B Baisor M Paino J Tamja S Adiguma T Wu L Rare L Bannon D Tielsch JM West KP Jr Alpers MP The influence of zinc supplementation on morbidity due to *Plasmodium falciparum*: a randomized trial in preschool children in Papua New Guinea Am J Trop Med Hyg 2000 62 663 669 11304051 10.4269/ajtmh.2000.62.663

[B12] Wintergerst ES Maggini S Hornig DH Immune-enhancing role of vitamin c and zinc and effect on clinical conditions Ann Nutr Metab 2006 50 2 85 94 10.1159/000090495 16373990

[B13] Wintergerst ES Maggini S Hornig DH Contribution of selected vitamins and trace elements to immune function Ann Nutr Metab 2007 51 4 301 323 10.1159/000107673 17726308

[B14] Shankar AH Prasad AS Zinc and immune function: the biological basis of altered resistance to infection Am J Clin Nutr 1998 68 2 447S 463S 9701160 10.1093/ajcn/68.2.447S

[B15] Caulfield LE Richard S Black RE Undernutrition as an underlying cause of malaria morbidity and mortality in children less than five years old Am J Trop Med Hyg 2004 71 Suppl 2 55S 63S 15331819

[B16] Mbugi EV Meijerink M Veenemans J Jeurink PV McCall M Olomi RM Shao JF Verhoef H Savelkoul HFJ Alterations in early cytokine-mediated immune response to Plasmodium falciperum infection in Tanzanian children with mineral deficiencies: a cross-sectional study Malaria In Press J 2010 in press 10.1186/1475-2875-9-130 PMC2881936 20470442

[B17] Jeurink PV Vissers YM Rappard B Savelkoul HFJ T cell responses in fresh and cryopreserved peripheral blood mononuclear cells: Kinetics of cell viability, cellular subsets, proliferation, and cytokine production Cryobiol 2008 57 2 91 103 10.1016/j.cryobiol.2008.06.002 18593572

[B18] Bell DR Wilson DW Martin LB False-positive results of a Plasmodium falciparum histidine-rich protein 2-detecting malaria rapid diagnostic test due to high sensitivity in a community with fluctuating low parasite density Am J Trop Med Hyg 2005 73 1 199 203 16014858

[B19] Wongsrichanalai C Barcus MJ Muth S Sutamihardja A Wernsdorfer WH A Review of Malaria Diagnostic Tools: Microscopy and Rapid Diagnostic Test (RDT) Am J Trop Med Hyg 2007 77 6 Suppl 119 127 18165483

[B20] Ponnudurai T Meuwissen JH Leeuwenberg AD Verhave JP Lensen AH The production of mature gametocytes of Plasmodium falciparum in continuous cultures of different isolates infective to mosquitoes Trans R Soc Trop Med Hyg 1982 76 2 242 250 10.1016/0035-9203(82)90289-9 7048650

[B21] Rivadeneira EM Wasserman M Espinal CT Separation and Concentration of Schizonts of Plasmodium falciparum by Percoll Gradients1 J Eukary Microbiol 1983 30 2 367 370 10.1111/j.1550-7408.1983.tb02932.x 6313915

[B22] Ryan A Analysis of Blood Serum on the Liberty Series II ICP-AES with the axially-viewed plasma ICP-24: Varian Inc 1998 1 9

[B23] Yssel H De Vries JE Koken M Van Blitterswijk W Spits H Serum-free medium for generation and propagation of functional human cytotoxic and helper T cell clones J Immunol Methods 1984 72 1 219 227 10.1016/0022-1759(84)90450-2 6086760

[B24] Phillips JH Hori T Nagler A Bhat N Spits H Lanier LL Ontogeny of human natural killer (NK) cells: fetal NK cells mediate cytolytic function and express cytoplasmic CD3εδ proteins J Exp Med 1992 175 4 1055 1066 10.1084/jem.175.4.1055 1372642 PMC2119193

[B25] Spits H Yssel H Terhorst C de Vries JE Establishment of human T lymphocyte clones highly cytotoxic for an EBV transformed B cell line in serum-free medium: isolation of clones that differ in phenotype and specificity J Immunol 1982 128 95 99 6274958

[B26] O'Dea KP Pasvol G Optimal tumor necrosis factor induction by *Plasmodium falciparum *requires the highly localized release of parasite products Infect Immun 2003 71 6 3155 3164 10.1128/IAI.71.6.3155-3164.2003 12761094 PMC155712

[B27] Walther M Woodruff J Edele F Jeffries D Tongren JE King E Andrews L Bejon P Gilbert SC De Souza JB Sinden R Hill AV Riley EM Innate immune responses to human malaria: heterogeneous cytokine responses to blood-stage *Plasmodium falciparum *correlate with parasitological and clinical outcomes J Immunol 2006 177 8 5736 5745 17015763 10.4049/jimmunol.177.8.5736

[B28] Waterfall M Black A Riley E γδ+ T Cells Preferentially Respond to Live Rather than Killed Malaria Parasites Infect Immun 1998 66 5 2393 2398 9573139 10.1128/iai.66.5.2393-2398.1998 PMC108213

[B29] Scholzen T Gerdes J The Ki-67 protein: from the known and the unknown J Cell Physiol 2000 182 3 311 322 10.1002/(SICI)1097-4652(200003)182:3<311::AID-JCP1>3.0.CO;2-9 10653597

[B30] Theander TG Defence mechanisms and immune evasion in the interplay between the humane immune system and Plasmodium falciparum Dan med bull 1992 39 1 49 63 1563295

[B31] Good MF Development of immunity to malaria may not be an entirely active process Parasite Immunol 1995 17 2 55 59 10.1111/j.1365-3024.1995.tb00966.x 7761108

[B32] Nylen S Mortberg U Kovalenko D Satti I Engstrom K Bakhiet M Akuffo H Differential induction of cellular responses by live and dead Leishmania promastigotes in healthy donors Clin Exp Immunol 2001 124 1 43 53 10.1046/j.1365-2249.2001.01501.x 11359441 PMC1906023

[B33] Prasad AS Effects of Zinc Deficiency on Th1 and Th2 Cytokine Shifts J Infect Dis 2000 182 S1 S62 S68 10.1086/315916 10944485

[B34] Beck FWJ Prasad AS Kaplan J Fitzgerald JT Brewer GJ Changes in cytokine production and T cell subpopulations in experimentally induced zinc-deficient humans Am J Physiol 1997 272 1002 1007 10.1152/ajpendo.1997.272.6.E1002 9227444

[B35] Sandstead HH Prasad AS Penland JG Beck FWJ Kaplan J Egger NG Alcock NW Carroll RM Ramanujam VMS Dayal HH Rocco CD Plotkin RA Zavaleta AN Zinc deficiency in Mexican American children: influence of zinc and other micronutrients on T cells, cytokines, and antiinflammatory plasma proteins Am J Clin Nutr 2008 88 4 1067 1073 18842795 10.1093/ajcn/88.4.1067

[B36] Ibs K-H Rink L Zinc-Altered Immune Function J Nutr 2003 133 5 1452S 1456S 12730441 10.1093/jn/133.5.1452S

[B37] Troye-Blomberg M Weidanz WP van der Heyde HC Wahlgren M, Perlmann P Amsterdam The role of T-cells in immunity to malaria and the pathogenesis of disease Malaria: Molecular and Clinical Aspects 1999 Harwood Academic 403 438

[B38] Lucey DR Clerici M Shearer GM Type 1 and type 2 cytokine dysregulation in human infectious, neoplastic, and inflammatory diseases Clin Microbiol Rev 1996 9 4 532 562 8894351 10.1128/cmr.9.4.532 PMC172909

[B39] Nyakeriga AM Williams TN Marsh K Wambua S Perlmann H Perlmann P Grandien A Troye-Blomberg M Cytokine mRNA expression and iron status in children living in a malaria endemic area Scand J Immunol 2005 61 4 370 375 10.1111/j.1365-3083.2005.01573.x 15853921

[B40] Nyakeriga AM Troye-Blomberg M Dorfman JR Alexander NlD Back R Kortok M Chemtai AK Marsh K Williams TN Iron Deficiency and Malaria among Children Living on the Coast of Kenya J Infect Dis 2004 190 3 439 447 10.1086/422331 15243915

[B41] Hershko C Mechanism of iron toxicity Food Nutr Bull 2007 28 4 Suppl S500 S509 18297888 10.1177/15648265070284S403

[B42] Weiss G Iron and immunity: a double-edged sword Eur J Clin Invest 2002 32 Suppl 1 S70 S78 10.1046/j.1365-2362.2002.0320s1070.x 11886435

[B43] Fritsche G Larcher C Schennach H Weiss G Regulatory Interactions between Iron and Nitric Oxide Metabolism for Immune Defense against Plasmodium falciparum Infection J Infect Dis 2001 183 9 1388 1394 10.1086/319860 11294671

[B44] Weiss G Thuma PE Mabeza G Werner ER Herold M Gordeuk VR Modulatory potential of iron chelation therapy on nitric oxide formation in cerebral malaria J Infect Dis 1997 175 1 226 230 8985227 10.1093/infdis/175.1.226

[B45] Black RE Therapeutic and preventive effects of zinc on serious childhood infectious diseases in developing countries Am J Clin Nutr 1998 68 2 476S 479S 9701163 10.1093/ajcn/68.2.476S

[B46] Black RE Black RE, Fleischer K Consequences of zinc de-ficiency on human health and alternatives for programmatic intervention Public Health Issues in Infant and Child Nutrition 2002 Philadelphia: Vevey/Lippincott 97 106

[B47] Keen CL Gershwin ME Zinc deficiency and immune function Annu Rev Nutr 1990 10 415 431 10.1146/annurev.nu.10.070190.002215 2200472

[B48] Muller O Becher H van Zweeden AB Ye Y Diallo DA Konate AT Gbangou A Kouyate B Garenne M Effect of zinc supplementation on malaria and other causes of morbidity in west African children: randomised double blind placebo controlled BMJ 2001 322 7302 1 6 10.1136/bmj.322.7302.1567 11431296 PMC33513

[B49] Zeba A Sorgho H Rouamba N Zongo I Rouamba J Guiguemde R Hamer D Mokhtar N Ouedraogo J-B Major reduction of malaria morbidity with combined vitamin A and zinc supplementation in young children in Burkina Faso: a randomized double blind trial Nutr J 2008 7 1 7 10.1186/1475-2891-7-7 18237394 PMC2254644

[B50] Sterne JAC Smith GD Sifting the evidence--what's wrong with significance tests? BMJ 2001 322 226 231 10.1136/bmj.322.7280.226 11159626 PMC1119478

[B51] Krzych U Lyon JA Jareed T Schneider I Hollingdale MR Gordon DM Ballou WR T lymphocytes from volunteers immunized with irradiated Plasmodium falciparum sporozoites recognize liver and blood stage malaria antigens J Immunol 1995 155 8 4072 4077 7561118

[B52] Roestenberg M McCall M Hopman J Wiersma J Luty AJF van Gemert GJ van de Vegte-Bolmer M van Schaijk B Teelen K Arens T Spaarman L de Mast Q Roeffen W Snounou G Renia L Ven van der A Hermsen CC Sauerwein R Protection against a Malaria Challenge by Sporozoite Inoculation N Engl J Med 2009 361 5 468 477 10.1056/NEJMoa0805832 19641203

